# Reductive Charge Transfer through an RNA Aptamer

**DOI:** 10.1002/anie.202009430

**Published:** 2020-10-13

**Authors:** Jennifer Frommer, Sabine Müller

**Affiliations:** ^1^ Institute for Biochemistry University Greifswald Felix Hausdorff Str. 4 17487 Greifswald Germany; ^2^ present address: School of Chemistry University of Birmingham Edgbaston Birmingham B15 2TT UK

**Keywords:** charge transfer, fluorescence quenching, optical spectroscopy, pyrene-modified RNA, RNA aptamers

## Abstract

The transfer of charges through double helical DNA is a very well investigated bioelectric phenomenon. RNA, on the contrary, has been less studied in this regard. The few available data report on charge transfer through RNA duplex structures mainly composed of homonucleotide sequences. In the light of the RNA world scenarios, it is an interesting question, if charge transfer can be coupled with RNA function. Functional RNAs however, contain versatile structural motifs. Therefore, electron transport also through non‐Watson–Crick base‐paired regions might be required. We here demonstrate distance‐dependent reductive charge transfer through RNA duplexes and through the non‐Watson–Crick base‐paired region of an RNA aptamer.

## Graphical abstract

RNA charge transfer: Reductive charge transfer through regular RNA duplexes and through a RNA aptamer is demonstrated. Charge transfer is induced by photochemical activation of 5‐dimethylaminopyreneuridine, incorporated in RNA as an electron donor. Charge transfer proceeds in a distance‐dependent manner and was monitored by fluorescence quenching and dehalogenation of 5‐bromodeoxyuridine, used as an electron trap.

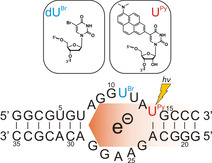

The strictly ordered structure of the DNA double helix has proven to be capable of transferring charges in the form of an electron over long distances.^[1]^ This discovery, made in 1993, opened the field of DNA charge transfer. Since then, the conductivity of DNA has been demonstrated in numerous charge transfer experiments.^[2]^ In addition to the biological relevance, those studies revealed important information on the sequence and distance dependence of the charge transfer and on various involved mechanisms.[^2a^, ^2b^] There are two different types of charge‐transfer reactions known, oxidative hole transfer and reductive electron transfer,^[3]^ the former being the more investigated phenomenon. The structure of the conductive DNA proofed to be extremely important, as mismatches and lesions were shown to cause loss of conductivity.^[4]^ Therefore, the highly ordered π‐stacking of the DNA duplex has been seen as a requirement for successful short as well as long range charge transfer.^[5]^ In the case of reductive charge transfer studies, the electron is transferred through a superexchange or hopping mechanism in a distance dependent manner.^[2c]^ The DNA sequence plays a major role, as the four nucleobases have different reduction potentials and affect the charge transfer mechanism.^[6]^ The so far available data on reductive RNA charge transfer result from studies on RNA duplex structures composed of homonucleotide sequences to ensure unhindered transfer of the electron,^[7]^ or were performed on hybrid duplexes of RNA with DNA or LNA counter strands.^[8]^ Over the past two decades, RNA has become a popular molecule as it is involved in numerous cellular functions, and moreover, is the fundamental player as functional molecule in the RNA world hypothesis.^[9]^ Therefore, it is a challenging question if RNA function may be coupled with charge transfer. In contrast to DNA, RNA is composed of versatile molecular structures, including loops, bulges or helical junctions, rather than of regular base‐paired helical regions. Hence, we set out to study reductive charge transfer not only through RNA duplexes, but in addition through a non‐Watson–Crick base paired region of an RNA aptamer.

For our studies we chose the flavine mononucleotide (FMN) aptamer, which was previously identified by in vitro selection. It is a rather small RNA structure comprising an 11 nucleotide bulge segment as the binding site for FMN,^[10]^ and it has been used in a number of studies for allosteric regulation of ribozyme activity.^[11]^ A particular attractive feature of FMN is its redox behavior. Upon reduction of the isoalloxazine ring, the molecular shape of FMN is supposed to change from planar to roof‐like. In previous work, we have used this characteristic of FMN to regulate activity of a FMN dependent hairpin aptazyme in a reversible manner.^[11c]^ FMN binds to the aptamer in its stable oxidized state, whereas reduction is associated with a change in the molecular shape of FMN and loss of binding capacity. If reductive charge transfer through the aptamer is possible, coupling of charge transfer with the FMN redox activity might become a, although highly ambitious, yet realistic vision to be further followed. In our previous work, we have used direct chemical or electrochemical reduction of FMN.[^11c^, ^11d^] In the envisioned system, suitable electron donors incorporated in the aptamer structure might deliver, upon photochemical activation, the electrons for reduction of FMN.

We started our investigation with a series of model duplexes and aptamer structures as shown in Figure 1. 5‐Dimethylaminopyreneuridine (5DMAPyU), which we have shown recently being an excellent electron donor when incorporated in RNA,^[12]^ was used for irradiation induced delivery of an electron into the RNA duplex or aptamer. Since reductive charge transfer occurs in competition to fluorescence, achievement of the charge separated state of the donor as prerequisite for charge transfer can be observed by fluorescence quenching.^[13]^ In addition, we used 5‐Bromodeoxyuridine (5BrdU) as electron trap, to observe distance and direction dependent charge transfer by 5BrdU dehalogenation.[^7a^, ^8^, ^14^] In the FMN aptamer, three different uridine residues were chosen for substitution with the electron donor 5DMAPyU. A11, playing a less important role in the aptamer structure,^[10]^ was chosen as a suitable site for 5BrdU as electron acceptor (Figure 1). All RNAs were synthesized by solid phase synthesis.

**Figure 1:**
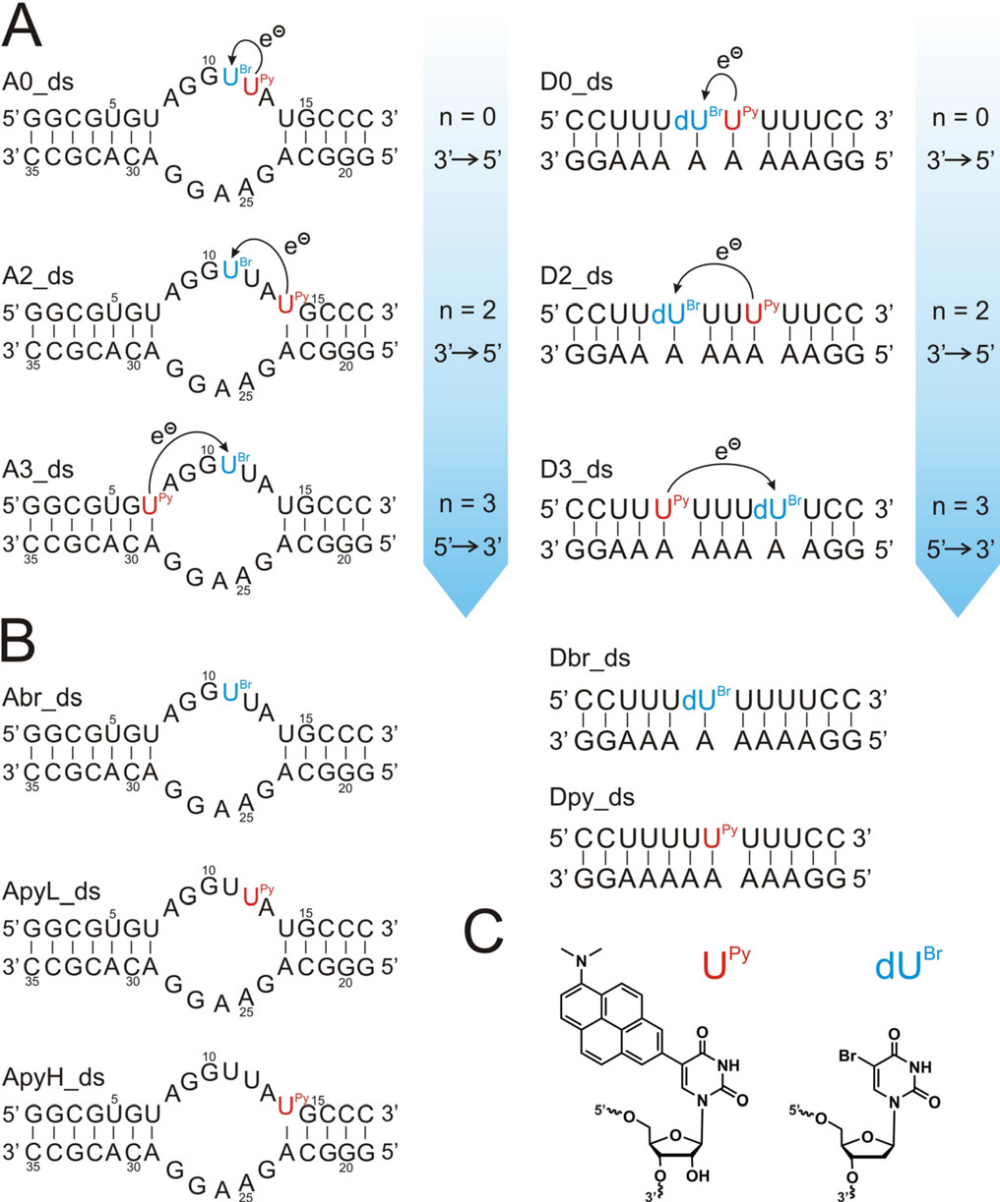
Model systems for RNA charge transfer studies. A) Aptamers and duplexes with electron donor (red) and electron acceptor (blue). B) Reference systems containing either the donor or the acceptor. C) Structure of 5DMAPyU (used as electron donor) and 5BrdU (used as electron acceptor).

First, we characterized our models regarding the influence of the pyrene moiety on duplex and aptamer stability. It has been shown previously that pyrene modifications attached to the sugar residue differently affect DNA and RNA duplexes,^[15]^ which would also play a crucial role for the charge transfer mechanism.^[7b]^ In DNA duplex structures, intercalation of the pyrene moiety was observed, whereas in RNA duplexes, it was found being oriented towards the major groove.^[15]^ Melting point analysis of our pyrene modified RNA duplexes and aptamers in sodium phosphate buffer (Na‐P_i_, pH 7) showed a differing impact of the pyrene moiety on the duplex versus aptamer stability (Table 1). The inserted pyrene slightly destabilizes all RNA duplexes, whereas the influence of the pyrene moiety on the aptamers **ApyL_ds**, **A0_ds** and **ApyH_ds** was found to be more stabilizing (Table 1). This may be a result of pyrene interacting with nucleobases in the bulge via hydrophobic stacking, similar to FMN when bound to the aptamer.^[10]^ However, if located in the helical region of the aptamer **A2_ds**, stability was comparable as measured for the non‐modified aptamer. There was no clear melting point detectable for **A3_ds**, instead multiple transitions were observed (see Supporting Information, Figure S1).

**Table 1:** Melting points (*T*_m_) of duplex and aptamer sequences.

	**X**=5DMAPyU, **Y**=5BrdU	Duplex^[a]^
	sequence	*T_m_* [°C]
**12**	5′ CCU UUU UUU UCC 3′	42.0±0.0
**Dpy**	5′ CCU UUU **X**UU UCC 3′	33.3±0.6
**D0**	5′ CCU UU**Y X**UU UCC 3′	32.4±0.6
**D2**	5′ CCU U**Y**U U**X**U UCC 3′	32.4±0.6
**D3**	5′ CCU U**X**U UU**Y** UCC 3′	32.4±1.2

	X=5DMAPyU, Y=5BrdU	Aptamer′^[b]^
	sequence	*T_m_* [°C]
**18**	5′ GGC GUG UAG GUU AUG CCC 3′	51.5±0.7^[c]^
**ApyL**	5′ GGC GUG UAG GU**X** AUG CCC 3′	54.6±0.8^[c]^
**A0**	5′ GGC GUG UAG G**YX** AUG CCC 3′	53.0±0.0^[c]^
**A2**	5′ GGC GUG UAG G**Y**U A**X**G CCC 3′	50.3±0.4^[c]^
**ApyH**	5′ GGC GUG UAG GUU A**X**G CCC 3′	53.1±1.3^[c]^
**A3**	5′ GGC GUG **X**AG G**Y**U AUG CCC 3′	n.d.^[c]^

[a] upon hybridisation with counter strand 5′ GGA AAA AAA AGG 3′; [b] upon hybridisation with counter strand 5′ GGG CAG AAG GAC ACG CC 3′; [c] measured in duplicates.

Another interesting observation was made through UV/Vis measurements. The pyrene specific absorbance between 330 and 400 nm of the RNA duplex structures showed differently pronounced first maxima at 347 nm (Figure S2). The extent of this first maximum appears to be dependent on the exact location of the electron acceptor and can be related to the achievement of the charge separated state of 5DMAPyU. Such differences in the absorbance pattern were not seen in previous studies with the sugar‐bound pyrene moiety.^[7a]^ The spectra of the aptamer structures also show different absorbance patterns, which are likely caused by the diverse RNA environment of helical versus loop structures (Figure S3).

For the investigation of charge transfer, we first looked at quenching of the pyrene fluorescence^[7a]^ in dependence on the presence and distance of the electron acceptor 5BrdU (Figure 2 A). As mentioned above, charge transfer competes with fluorescence, and requires the generation of a charge separated state of the excited donor moiety. As a consequence, the fluorescence intensity is decreased, and this can be taken as direct evidence for successful charge transfer.^[13]^ Fluorescence emission spectra of all duplexes were recorded at 2.5 μM duplex concentration in Na‐P_i_, at an excitation wavelength of 350 nm (Figure 2 A). Duplex **Dpy_ds**, only equipped with the electron donor 5DMAPyU, was used as reference. A quantum yield of Φ=0.0006 was determined for the pyrene fluorescence in **Dpy_ds** (Table S2), which is significantly smaller than that determined for a pyrene moiety attached to the sugar backbone of a RNA homoduplex used in previous studies.^[7a]^ We had already shown recently, that for the modified uridine derivative used here, where 1‐dimethylaminopyrene is attached to the nucleobase, strong fluorescence quenching occurs,^[12]^ implying that the charge separated state is achieved nearly completely via a TICT mechanism.^[16]^

**Figure 2:**
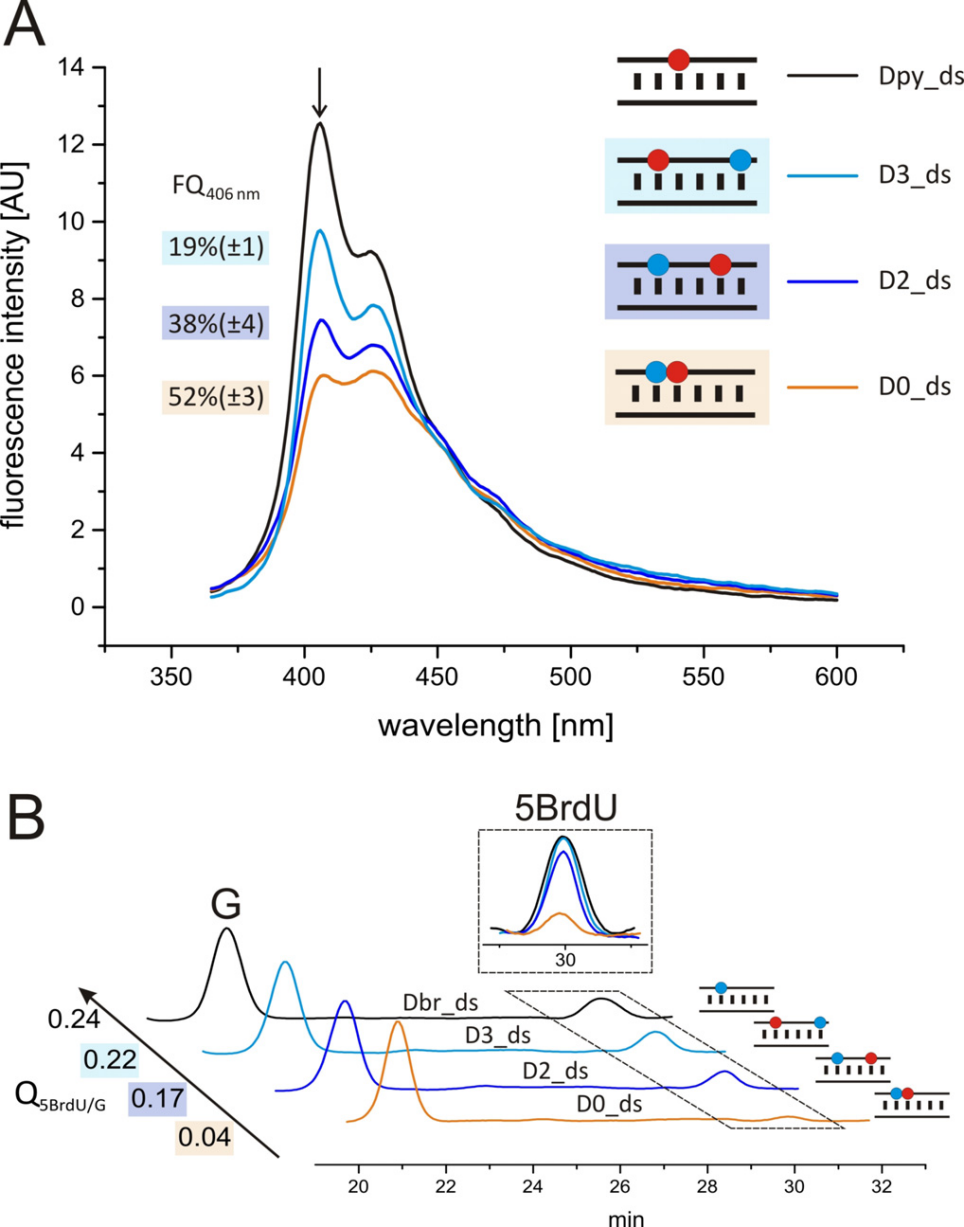
Reductive charge transfer through RNA duplex structures containing the electron donor 5DMAPyU (red) and the electron acceptor 5BrdU (blue). A) Fluorescence spectra (excitation at 350 nm) of RNA duplexes (Dpy_ds serving as reference). B) RP‐HPLC analysis of RNA duplexes after irradiation at 365 nm for 40 min and enzymatic digestion. Observed is the degradation of the electron trap 5BrdU upon irradiation. The peak area of 5BrdU was set in relation to the peak area of G for calculation of Q_5BrdU/G_ (see Supporting Information).

Fluorescence values at 406 nm were used to quantify fluorescence quenching (FQ) in the different duplexes (Figure 2 A). The resulting FQ values show the reduction in the fluorescence intensity for each investigated duplex D**n**_ds (with n=number of nucleotides that separate donor and acceptor) in relation to the reference **Dpy_ds**. Fluorescence quenching was 52 % for *n*=0 (in **D0_ds**), 38 % for *n*=2 (in **D2_ds**), and 19 % for *n*=3 (in **D3_ds**) (Figure 2 A). For the latter, it has to be taken into account that charge transfer occurs in the opposite direction as compared with **D0_ds** and **D2_ds**. This may also influence charge transfer efficiency, but independent on this, the general trend of decreased charge transfer with increasing distance between electron donor and acceptor is clearly visible.

Additional evidence for successful charge transport was provided by the dehalogenation assay. Transfer of an electron from the pyrene modified uridine derivative 5DMAPyU to the acceptor 5BrdU leads to very fast debromination via a radical mechanism.^[14]^ Thus, 5BrdU functions as electron trap. Debromination can be analyzed by RP‐HPLC, following the decomposing of 5BrdU. First, a 2.5 μM solution of RNA duplex **Dbr_ds** (only equipped with the electron acceptor 5BrdU, thus serving as reference) in Na‐P_i_, was irradiated at 365 nm for 40 min, followed by enzymatic digestion and analysis of the resulting nucleoside mixture by RP‐HPLC (Figure 2 B). Nucleoside and protein standards were used for identification of peaks (Figure S4).^[7b]^

The signal for 5BrdU is clearly visible at a retention time of about 30 min. For internal standardization, the area of the 5BrdU peak was set in relation to the peak area corresponding to guanosine, and the quotient (Q_5BrdU/G_) was taken as a measure of the 5BrdU content in the individual samples. Guanosine was chosen, because it has the smallest reducibility of the four nucleobases,[^6b^, ^6c^] and therefore the smallest susceptibility to reductive damage upon charge transfer, which cannot be completely excluded. On the other hand, guanosine is most easily oxidized, which might lead also to oxidative damage upon irradiation. However, previous studies have shown, that oxidation of guanosine by DMAPy‐linked deoxynucleosides as the built‐in electron donor is rather unlikely.^[17]^ After irradiation of duplexes **D0_ds**, **D2_ds** and **D3_ds**, followed by enzymatic digestion and RP‐HPLC analysis, reduction of the 5BrdU peak area in relation to the peak area of G is clearly visible, thus indicating debromination of 5BrdU and thereby confirming successful charge transfer. Moreover, as already seen in the fluorescence quenching assay, charge transfer occurs in a distance dependent manner, as Q_5BrdU/G_ is most significantly reduced for **D0_ds**, followed by **D2_ds** and **D3_ds** in relation to **Dbr_ds** (Figure 2 B).

After having successfully shown distant dependent charge transfer through RNA duplexes with 5DMAPyU as electron donor, we were interested in investigating this phenomenon with the FMN aptamer. Looking at the recorded spectra in the fluorescence quenching assay, the fluorescence intensity appears to be strongly dependent on the specific location of 5DMAPyU as electron donor, as seen with aptamer structures **ApyL_ds** and **ApyH_ds**, used as references (Figure 3 A). Fluorescence quenching of 24 % (for **A0_ds**) and 19 % (for **A2_ds**) in relation to the reference aptamers **ApyL_ds** and **ApyH_ds** is clearly visible, indicating successful charge transfer through both aptamer structures.

**Figure 3:**
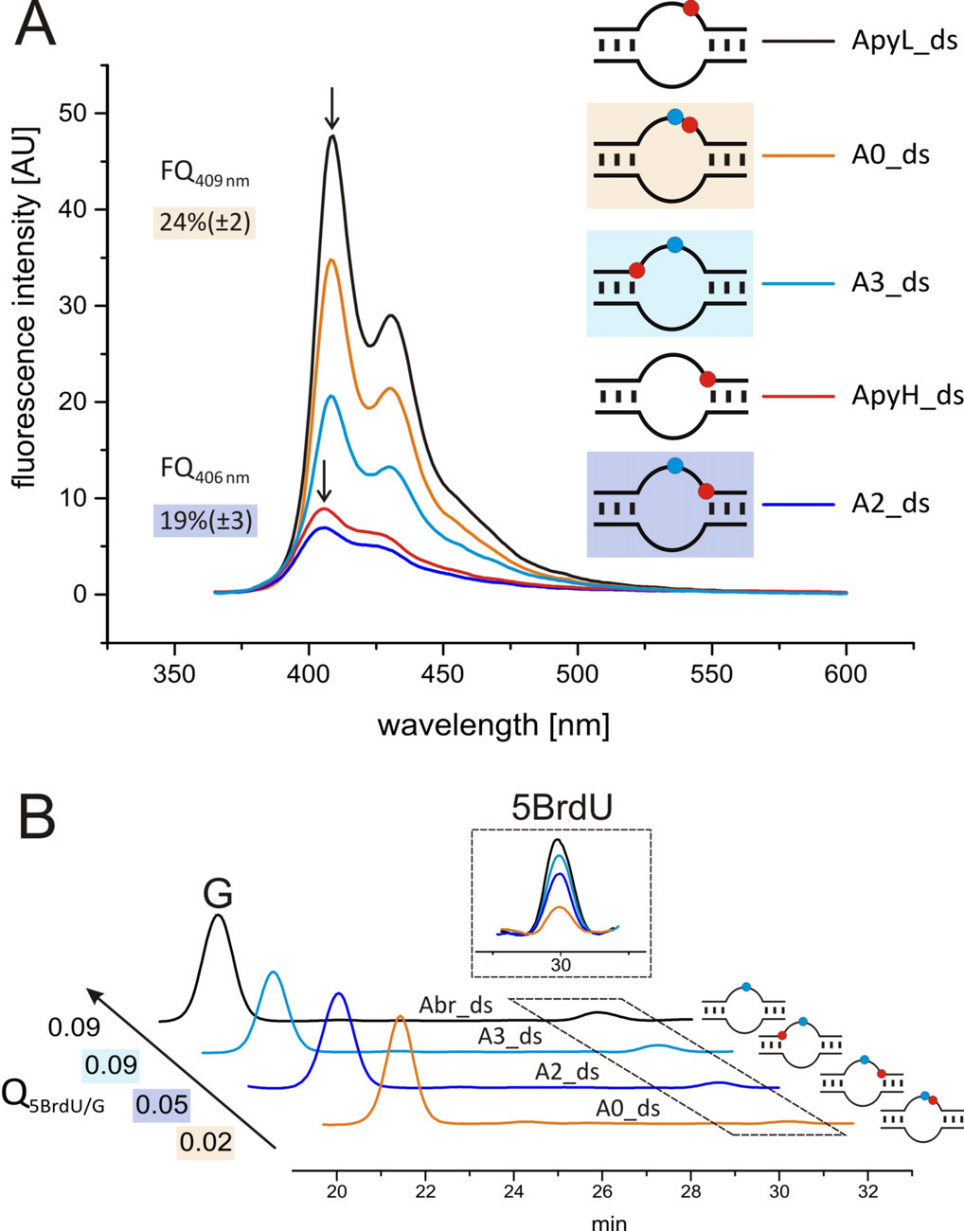
Reductive charge transfer through RNA aptamers containing the electron donor 5DMAPyU (red) and the electron acceptor 5BrdU (blue). A) Fluorescence spectra (excitation at 350 nm) of the RNA aptamers (ApyL_ds and ApyH_ds serving as references). B) RP‐HPLC analysis of RNA aptamers after irradiation at 365 nm for 40 min and enzymatic digestion. Observed is the degradation of the electron trap 5BrdU upon irradiation. The peak area of 5BrdU was set in relation to the peak area of G for calculation of Q_5BrdU/G_ (see Supporting Information).

The observed fluorescence of **A3_ds** is significantly higher compared to **ApyH_ds**, which, with 5DMAPyU in the helical region, is the closest system to serve as standard. The relatively high fluorescence observed for **A3_ds** implies that excess electron transfer does not occur. This result is confirmed by the results of the dehalogenation assay (see below), and is most likely a consequence of the nucleotide sequence in the neighborhood of 5DMAPyU in **A3_ds**.^[18]^

Whereas in **A2_ds**, charge transfer occurs through an adenosine and uridine, both nucleosides having a medium to high reduction potential, in **A3_ds** the electron needs to be transferred through an adenosine and two guanosines (Figure 1). As mentioned above, guanosine has the lowest reduction potential, and the electron donating force of 5DMAPyU is presumably not sufficient to reduce G.^[17]^ As a consequence, the driving force for achievement of a charge‐separated state is significantly reduced, which would result in a stronger dominance of the fluorescence in competition to charge transfer.[^6a^, ^18^] Thus, transfer of an excess electron via a hopping mechanism can be excluded for **A3_ds**.[^6a^, ^18^] There is still the possibility that charge transfer occurs via a super‐exchange mechanism using long irradiation times, albeit the results of the dehalogenation assay imply that this is also not the case.

As for the RNA duplexes, aptamers were irradiated at 365 nm for 40 min to follow charge transfer in the dehalogenation assay. Again, the guanosine peak area was used for internal standardization. Note, that the Q_5BrdU/G_ values for the aptamer RNAs differ from the values of the duplexes, since the aptamer sequences have a higher guanosine content. For aptamers **A2_ds** and **A0_ds**, the expected reduction of the 5BrdU peak area and consequently a decrease of Q_5BrdU/G_ (0.05 for **A2_ds** and 0.02 for **A0_ds**) in comparison to the **Abr_ds** reference (Q_5BrdU/G_=0.09) is observed (Figure 3 B). This is in good agreement with the results of the fluorescence quenching assay and confirms, that charge transfer occurs in a distant dependent manner.

Owing to the long irradiation times used in the dehalogenation assay, the extent of 5BrdU degradation is remarkably similar in the duplex and aptamer structures, as can be seen when converting Q_5BrdU/G_ into percentage values, considering the differing guanosine content in both, duplexes and aptamers (Table S3).

The calculated Q_5BrdU/G_ value for **A3_ds** is the same as for the reference system **Abr_ds** (Q_5BrdU/G_=0.09). This implies that, upon irradiation of **A3_ds**, 5BrdU remains undamaged, which allows to conclude that transfer of an electron from the pyrene modified uridine derivative to 5BrdU did not occur. This is, as discussed above, most likely a consequence of the two guanosine residues between electron donor and acceptor, since charge transfer with donor and acceptor separated by 3 nts, as well as in 5′→3′ direction was clearly shown in the RNA duplexes (Figure 2).

Early investigations of intramolecular charge transfer processes identified a correlation of several factors how the interaction of donor and acceptor moieties lead to different transfer mechanisms. The electronic coupling, as well as the distance between the donor and acceptor moiety are crucial factors maintaining a certain type of charge transfer mechanism.^[19]^ The involved transfer mechanisms were classified as through‐bond and through‐space mechanism, whereas the electronic coupling is a sufficient condition leading to a charge separated state.^[19a]^ These transfer mechanisms were transferred to describe the charge transfer processes in nucleic acids, including the same rules of electronic coupling and distance dependency. The superexchange mechanism (Figure 4 A) is discussed to occur in short distances around 10 Å, and the hopping mechanism (Figure 4 B) to occur in long range distances of 25 Å and greater.[^2a^, ^2c^, ^19a^, ^19c^]

**Figure 4:**
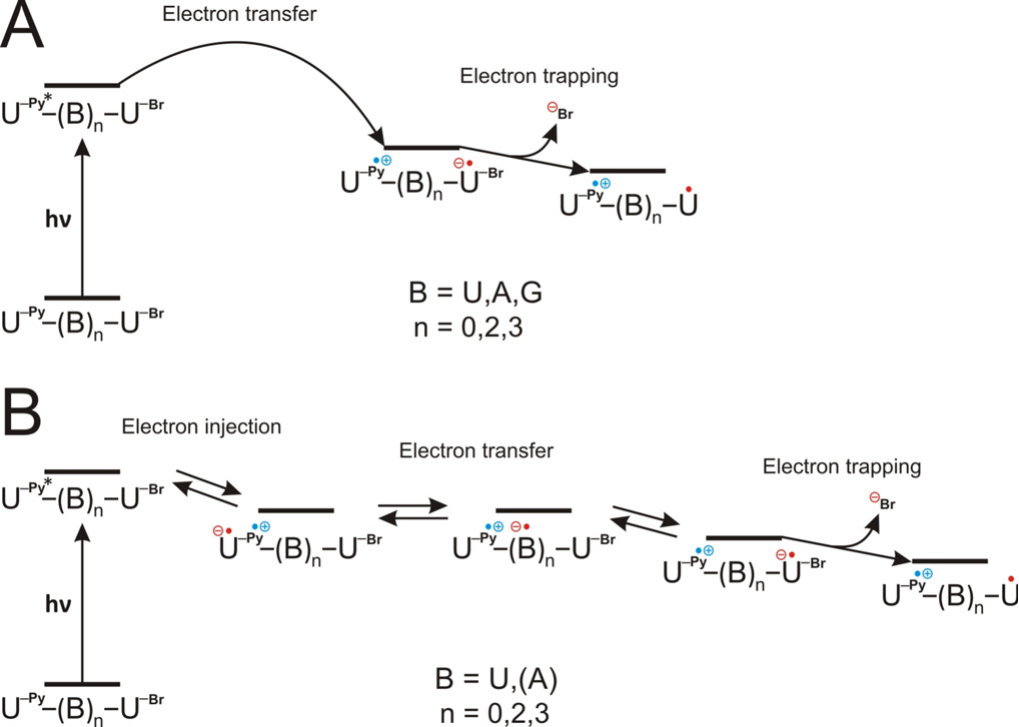
Reductive charge transfer mechanisms after the excitation of the DMAPy moiety. A) The excess electron is transferred in a single step via a superexchange mechanism, utilising the RNA as bridge between donor (DMAPyU) and acceptor (5BrdU). B) The excess electron is transferred in multiple steps via a hopping mechanism, involving the nucleotides between donor (DMAPyU) and final acceptor (5BrdU) as electron acceptors.

For DNA it was shown, that the mechanism of reductive charge transfer is sequence‐dependent and it was suggested, that intra‐strand electron tunneling can take place, if guanosines were present in the sequence.[^6a^, ^18^] The requirement for a tunneling mechanism is an alternating sequence of guanosine and thymidine in the same strand, whereas multiple guanosine insertions lead to termination of the electron transfer.^[6a]^

These conclusions were drawn from experiments with DNA duplex structures, where unhindered electron transfer occurs merely if a fully matched DNA duplex without lesions is available.[^2a^, ^5a^]

The here described electron transfer distances in the RNA duplex for *n*=0, 2, 3 correspond to a distance of 2.8 Å, 8.4 Å and 11.2 Å (Table S4), making a superexchange mechanism possible, but do not exclude the hopping mechanism completely. The strong FQ effect of the donor moiety and nearly complete degradation of the electron trap in **D0_ds** make a superexchange mechanism very likely. On the other hand, the distances in the RNA aptamer structure are not clearly to obtain, since only the FMN bound structure is available. The A11 position folds out, to let the FMN ligand into the loop structure. Therefore, the FMN position within the FMN aptamer was used to obtain the distances between the donor and acceptor moiety (Figure S6). The distances for *n*=0, 2, 3 correspond to a distance of 5.0 Å, 10.4 Å and 11.7 Å in the aptamer region, which would explain the less efficient transfer rate of the excess electron for **A0_ds** and **A2_ds**.

Moreover, together with the above described similar degradation of 5BrdU in duplex and aptamer structures (in contrast to differing FQ values), a hopping mechanism is very likely for the aptamer structures, since obtaining a charge separated state in a multi‐step transfer reaction involves several equilibrium reactions with the possibility of a back electron transfer.^[20]^

However, the rather medium reduction potential of the adenosine nucleobase sets a rather high barrier for the hopping mechanism. The here used electron donor 5DMAPyU could play a crucial role, since the charge separated state is reached by a TICT mechanism.^[12]^ The involved electron donor geometry during excitation in combination with the aptamer architecture can have an impact on the charge transfer mechanism.[^19b^, ^19c^] Apart from our study reported herein, there are no data available yet for electron transfer through RNA structures of mixed sequence. To what extent the sequence and/or the specific structure is crucial for a successful electron transfer in RNA systems and which transfer mechanisms are involved, remains to be systematically elucidated in further experiments.

Independent of the results for the **A3_ds** aptamer, our results clearly show that reductive charge transfer in a distant dependent manner proceeds not only through regular RNA duplex structures, but also through an RNA aptamer. To the best of our knowledge, this is the first example of charge transport through a non‐Watson–Crick base paired RNA region. There is no structure of the FMN aptamer in the absence of the ligand available. However, the NMR structure in the presence of FMN shows that base stacking is a characteristic structural feature of the aptamer 11 nucleotide bulge region.^[10]^ Provided that base stacking also occurs in the absence of FMN, this could be a sufficient criterion for the observed transfer of an electron. The results reported herein are a first indication that charge transfer through RNA structures with specific functions is possible and thus pave the way towards design of more complex functional systems driven by the induced transport of electrons.

## Conflict of interest

The authors declare no conflict of interest.

## Supporting information

As a service to our authors and readers, this journal provides supporting information supplied by the authors. Such materials are peer reviewed and may be re‐organized for online delivery, but are not copy‐edited or typeset. Technical support issues arising from supporting information (other than missing files) should be addressed to the authors.

Supplementary
