# Supplementary material for: Reductive Charge Transfer through an RNA Aptamer
Source: Angew Chem Int Ed Engl. 2020 Oct 13;59(51):22999–3004. doi: 10.1002/anie.202009430 (PMC7756803; doi:10.1002/anie.202009430)
Supplement: Supplementary file 1 — Supplementary [file ANIE-59-22999-s001.pdf]

Angewandte Chemie
Eine Zeitschrift der Gesellschaft Deutscher Chemiker

Supporting Information

Reductive Charge Transfer through an RNA Aptamer

*Jennifer Frommer and Sabine Müller**

anie_202009430_sm_miscellaneous_information.pdf

Table of Contents

Table of Contents 1
Experimental Procedures 2
General 2
RNA preparation 2
Irradiation of the RNA samples 2
Enzymatic RNA digestion 2
RP-HPLC 2
Determination of $Q_{5BrdU/G}$ 3
Determination of quantum yields 3
Supplementary Figures and Tables 4
References 13
Author Contributions 13

Experimental Procedures

The synthesis of the 5DMAPyU phosphoramidite was described in our earlier work.[1]

General

Mass spectra were recorded on a Bruker microflex MALDI-TOF MS. UV/Vis absorption measurements were recorded with a JASCO V-650. Fluorescence measurements were performed with a JASCO FP-6500 spectrofluorometer. All spectra were recorded at 22 °C in Na–Pi with an RNA concentration of 2.5 µM. UV melting curves were determined using the Cary 100 spectrophotometer equipped with a temperature control unit (Varian), applying a heating/cooling rate of 0.2 °C/min in a temperature range from 10 °C or 15 °C to 90 °C. All optical spectroscopy was performed in triplet measurements, except the T_m determination of the aptamer sequences, which was done in duplicates. Na-P_i buffer was prepared to contain 20 mM sodium phosphate and 100 mM NaCl at pH 7. Phosphordiesterase (*Crotalus atrox*) was obtained from Sigma-Aldrich and Shrimp Alkaline Phosphatase from Affymetrix. All other reagents or chemicals and solvents were obtained as the highest commercially available grade and used without further purification.

RNA preparation

RNAs were synthesized on a Gene Assembler special DNA/RNA Synthesizer following the standard protocol for oligoribonucleotide chain assembly. Standard PAC-phosphoramidites as well as CPG supports were obtained from ChemGenes or Link Technologies. For removal of base and phosphate protecting groups and cleavage from the support, the synthesized RNAs were incubated with aqueous ammonia (32%)/ethanolic methylamine (8 M) (1:1, v/v) at 65 °C for 40 min., followed by incubation with TEA·3HF for 1.5 h at 55 °C for removal of the 2'-O-protecting groups. Purification of the synthesized RNA strands was performed by denaturing PAGE (12.5%, urea and acrylamide (acrylamide/bis-acrylamide 19:1) were purchased from Roth). Product containing bands were cut out, eluted from the gel with 0.3 M NaOAc, pH 6.5, and desalted via precipitation with ethanol. dsRNA for fluorescence and UV/Vis-measurements and for UV melting curves was prepared in Na-P_i buffer by mixing equimolar amounts of complementary strands to a final concentration of 2.5 µM. Hybridisation was promoted by heating the mixture at 90 °C for 2 min followed by slow cooling at room temperature for gradual annealing.

Irradiation of the RNA samples

The irradiation of the RNA samples was carried out using a concentration of 2.5 µM in Na-P_i buffer. The RNA solutions had been filled into quartz glass precision cuvettes (500 µl, path length 1 cm, Hellma Analytics) and were irradiated for 40 min with light of a wavelength of 365 nm (UV lamp). After irradiation, the RNA was precipitated by adding 300 vol% ethanol. Afterwards, the precipitated and dried RNA was digested enzymatically.

Enzymatic RNA digestion

The phosphodiesterase (*Crotalus atrox*, 0.01 U/mg) was freshly dissolved in H_2O before use. The reaction mixture was prepared by mixing 6 µl Tris/HCl buffer (40 mM Tris, 40 mM $MgCl_2$, pH 8.9) to 1 nmol of the respective RNA, followed by the addition of 3 µl of the phosphodiesterase solution (0.003 U/µl) and 1 µl of alkaline phosphatase (Shrimp Alkaline Phosphatase, 1 U/µl). The resulting reaction mixture was incubated at 37 °C for 24 h. The reaction was stopped by adding 100 µl water, followed by analysis via analytical RP-HPLC.

RP-HPLC

The digested RNA samples were run on Akta Purifier (Amersham Biosciences) using a Nucleosil 125/4 (120-5 C18) column from Macherey-Nagel with a column volume (CV) of 1.571 ml. The analytical RP-HPLC run was executed by using a flowrate of 0.5 ml/min with a solvent gradient consisting of buffer A (0.05 M TEAAc) and buffer B (0.1 M TEAAc, 30% MeCN). The used solvent gradient was: isocratic 1% (B) for 2 CV, linear gradient to 16% (B) in 12 CV, linear gradient to 100% (B) in 10 CV and isocratic 100% (B) for 4 CV.

The resulting nucleoside mixture was analyzed using nucleoside and protein standards. Inosine (I) was identified instead of adenosine, due to an adenosine deaminase-contamination of the phosphodiesterase.[2]

Determination of $Q_{5BrdU/G}$

The integration of the peak areas was carried out with Origin 8.0. The peak area of guanosine and 5BrdU were determined for each analytical RP-HPLC run. The quotient was generated by dividing the peak area of 5BrdU by the peak area of the respective guanosine.

Determination of quantum yields

Quantum yields were estimated using quinine sulfate in 0.1 N H_2SO_4 as reference following a standard procedure.[3] In order to avoid internal filter effects, the absorption of the sample and reference substance at the excitation wavelength was kept below 0.05.

Supplementary Figures and Tables

Table S1. Mass data of the synthesized duplex and aptamer sequences.

X = 5DMAPyU, **Y** = 5BrdU

	sequence	m/z [M+H]$^+$	calc. [M+H]$^+$
**Dpy**	5’ CCU UUU **X**UU UCC 3’	3854	3854
**D0**	5’ CCU UU**Y** **X**UU UCC 3’	3918	3917
**D2**	5’ CCU U**Y**U U**X**U UCC 3’	3918	3917
**D3**	5’ CCU U**X**U UU**Y** UCC 3’	3917	3917

X = 5DMAPyU, **Y** = 5BrdU

	sequence	m/z [M+H]$^+$	calc. [M+H]$^+$
**ApyL**	5’ GGC GUG UAG GU**X** AUG CCC 3‘	6013	6010
**A0**	5’ GGC GUG UAG G**YX** AUG CCC 3‘	6074	6072
**A2**	5’ GGC GUG UAG G**Y**U A**X**G CCC 3‘	6074	6072
**ApyH**	5’ GGC GUG UAG GUU A**X**G CCC 3‘	6020	6010
**A3**	5’ GGC GUG **X**AG G**Y**U AUG CCC 3‘	6084	6072

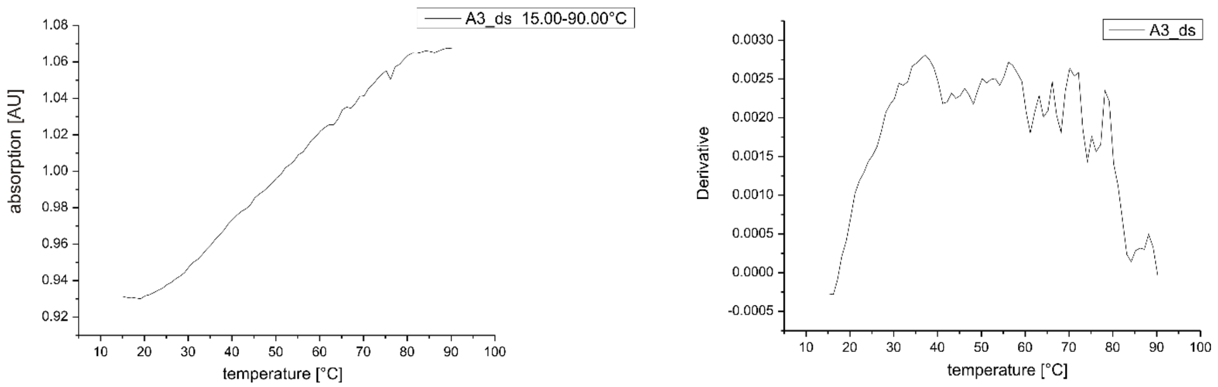

Figure S1. UV melting curve of A3_ds and the corresponding derivation, which lacks a clear maximum for melting point determination.

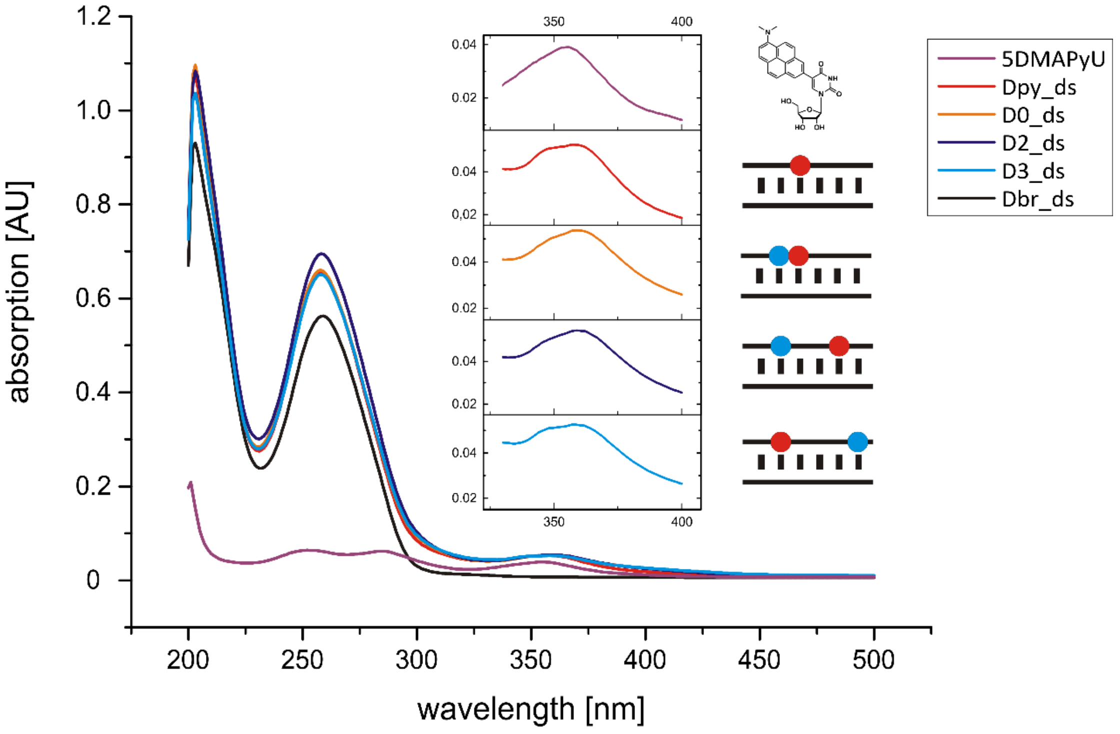

Figure S2. UV/Vis absorption spectra of 5DMAPyU in comparison to the 5DMAPyU modified RNA duplex sequences. All spectra were recorded at 22 °C at a concentration of 2.5 µM in Na-P_i for the RNAs and 1.5 µM for DMAPyU. The pyrene absorption range between 330 and 400 nm for each sample is magnified in the extract.

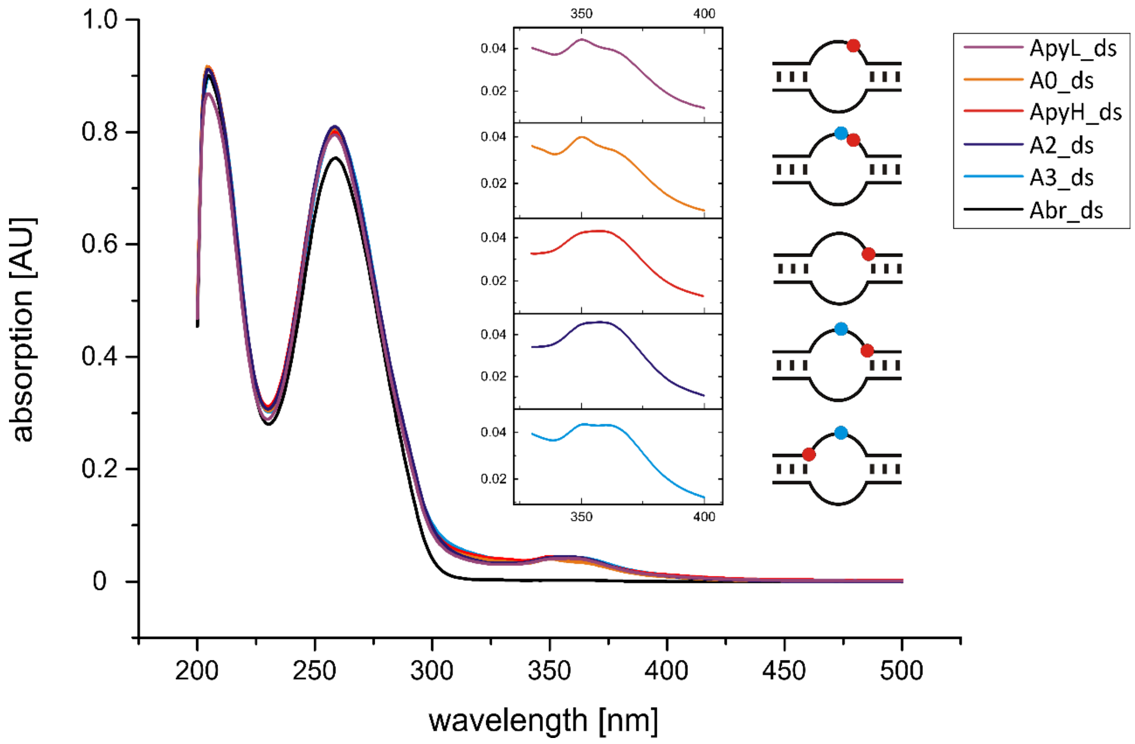

Figure S3. UV/Vis absorption spectra of the 5DMAPyU modified RNA aptamer sequences. All spectra were recorded at 22 °C at a concentration of 2.5 µM in Na-P$_i$. The pyrene absorption range between 330 and 400 nm for each sample is magnified in the extract.

Table S2. Fluorescence quantum yields of 5DMAPyU, Dpy_ds and the aptamer sequences ApyL_ds, ApyH_ds, A0_ds, A2_ds and A3_ds in Na-P$_i$.

**Nucleoside**	**Φ (NaP$_i$)**
5DMAPyU	0.001
**Duplex**	**Φ (NaP$_i$)**
Dpy_ds	0.0006
**Aptamer**	**Φ (NaP$_i$)**
ApyL_ds	0.0022
A0_ds	0.0018
ApyH_ds	0.0006
A2_ds	0.0004
A3_ds	0.0011

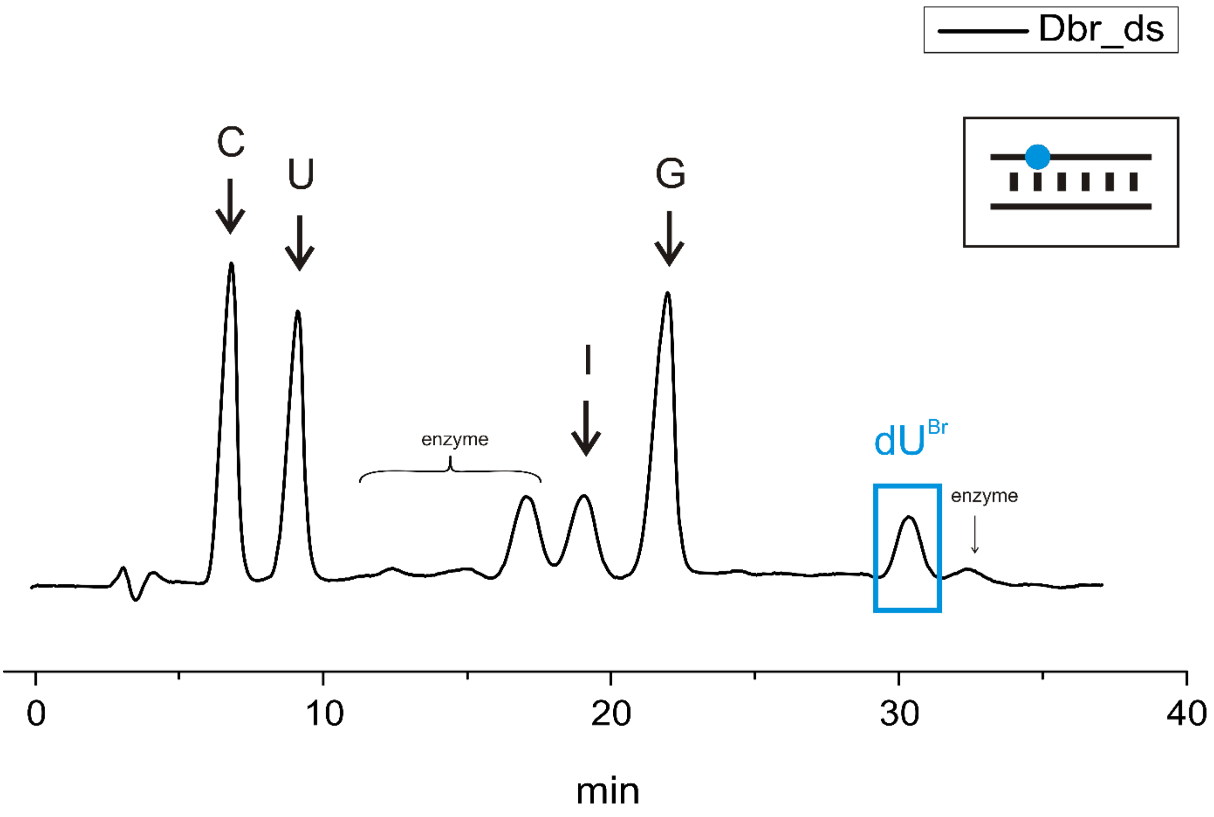

Figure S4. RP-HPLC run of the irradiated and digested RNA duplex Dbr_ds, containing only the electron acceptor 5BrdU.

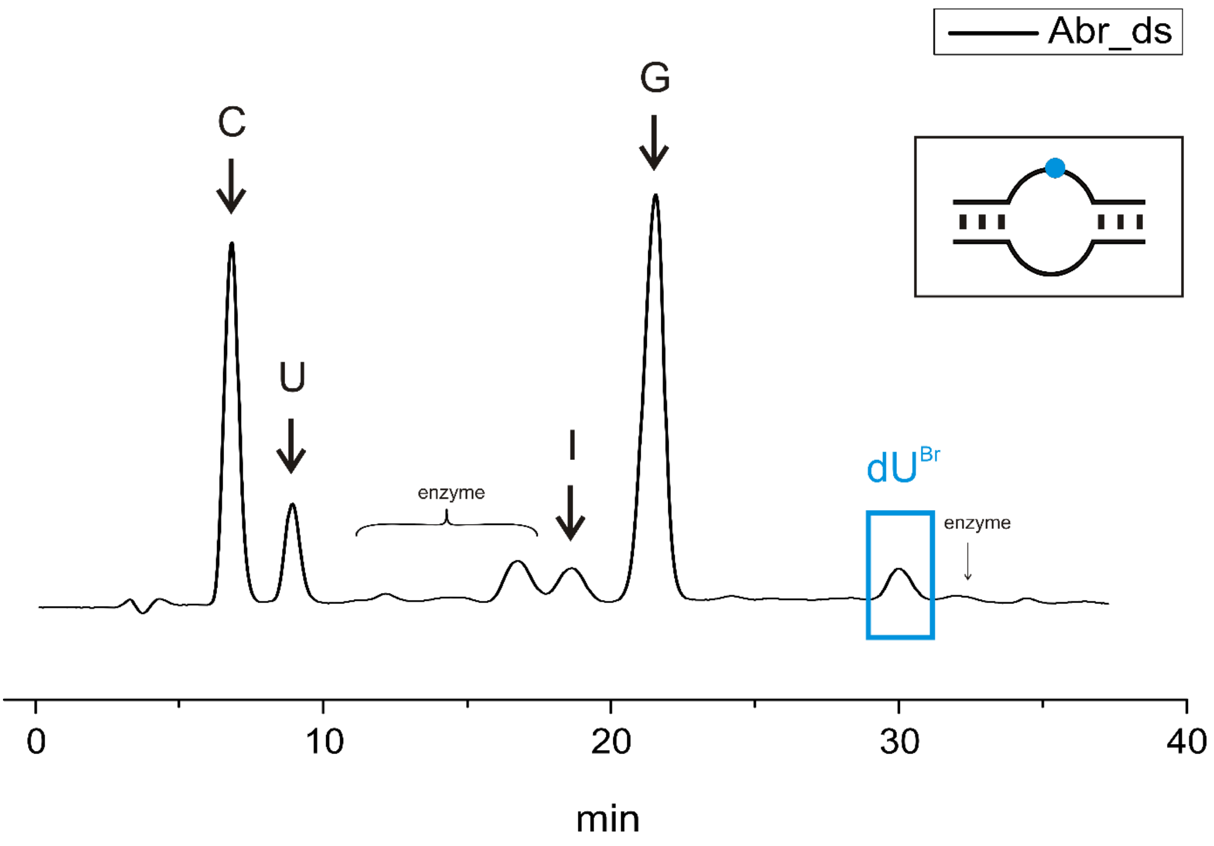

Figure S5. RP-HPLC run of the irradiated and digested RNA aptamer Abr_ds, containing only the electron acceptor 5BrdU.

Table S3. Comparison of the duplex and aptamer sequence $Q_{5BrdU/G}$ values.

Duplex structures	$Q_{5BrdU/G}$	%	Aptamer structures	$Q_{5BrdU/G}$	%
Dbr_ds	0.24	100	Abr_ds	0.09	100
D3_ds	0.22	86	A3_ds	0.09	100
D2_ds	0.17	67	A2_ds	0.05	58
D0_ds	0.04	17	A0_ds	0.02	26

Table S4. Comparison of the distances between the donor and acceptor moiety in the duplex and aptamer structure. The distances for the RNA Duplex are the average values in A-RNA. The distances for the RNA Aptamer were measured in PyMol (**Fig. S6**)

Nucleotides between donor and acceptor	RNA Duplex	RNA Aptamer
n	distance in Å	distance in Å
0	2.8	5
2	8.4	10.4
3	11.2	11.7

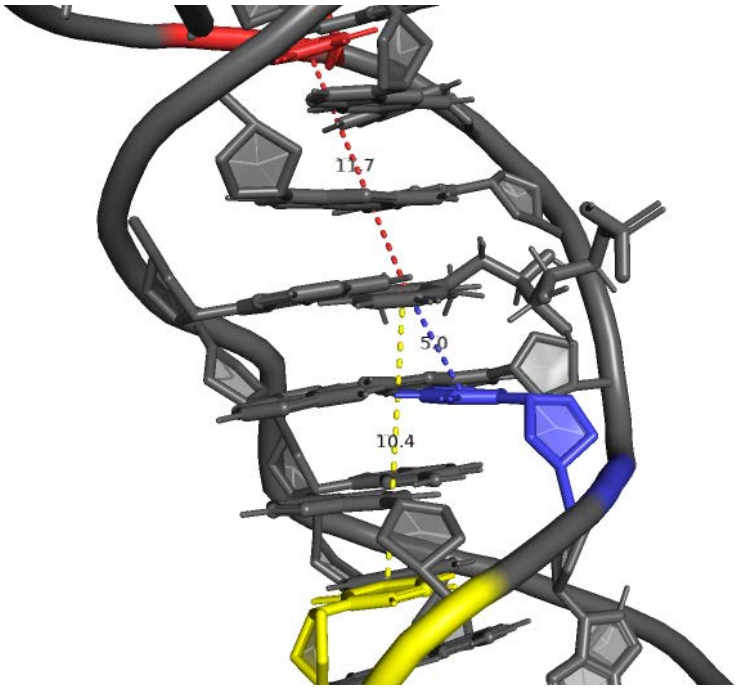

Figure S6. Structure of the FMN Aptamer [pdb: 1FMN**].** The FMN position within the FMN aptamer was used to obtain the distances between the donor and acceptor moiety for n = 0 (blue), 2 (yellow), 3 (red).

References

[1] J. Frommer, B. Karg, K. Weisz, S. Muller, Org Biomol Chem 2018, 16, 7663-7673.
[2] P. Cekan, S. T. Sigurdsson, JACS 2009, 131, 18054–18056
[3] J. R. Lakowicz, Principles of Fluorescence Spectroscopy, 3rd Edition, Springer US, **2006**, pp. 52-53.

Author Contributions

J.F. and S.M. planned the experiments. J.F. carried out the experiments and analysed the data. S.M. supervised the project. J.F. and S.M. wrote the manuscript.
